# An Electroanalytical Solution for the Determination of Pb^2+^ in Progressive Hair Dyes Using the Cork–Graphite Sensor

**DOI:** 10.3390/s22041466

**Published:** 2022-02-14

**Authors:** Thalita Medeiros Barros, Danyelle Medeiros de Araújo, Alana Tamires Lemos de Melo, Carlos Alberto Martínez-Huitle, Marco Vocciante, Sergio Ferro, Elisama Vieira dos Santos

**Affiliations:** 1 Laboratório de Eletroquímica Ambiental e Aplicada, Instituto de Química, Universidade Federal do Rio Grande do Norte, Lagoa Nova, Natal 59.072-900, RN, Brazil; thalita.medeiros.barros@hotmail.com (T.M.B.); danny@ccet.ufrn.br (D.M.d.A.); alanalemos1@hotmail.com (A.T.L.d.M.); carlosmh@quimica.ufrn.br (C.A.M.-H.); 2 Laboratório de Eletroquímica e Química Analítica, Programa de Pós Graduação em Ciências Naturais, Universidade do Estado do Rio Grande do Norte, Natal 59.610-210, RN, Brazil; 3 Department of Chemistry and Industrial Chemistry, University of Genova, 16146 Genova, Italy; marco.vocciante@unige.it; 4 Ecas4 Australia Pty Ltd., Mile End South, SA 5031, Australia

**Keywords:** cork, graphite electrode, electroanalysis, lead, progressive hair dyes

## Abstract

Lead is one of the most toxic metals for living organisms: once absorbed by soft tissues, it is capable of triggering various pathologies, subsequently bioaccumulating in the bones. In consideration of this, its detection and quantification in products for human consumption and use is of great interest, especially if the procedure can be carried out in an easy, reproducible and economical way. This work presents the results of the electroanalytical determination of lead in three different commercial products used as progressive hair dyes. Analyses were performed by cyclic voltammetry (CV) and differential pulse stripping voltammetry (DPSV) using a composite cork–graphite sensor in 0.5M H_2_SO_4_ solution or 0.1M acetate buffer (pH 4.5), in the presence and absence of hair dye samples. The H_2_SO_4_ solution gave better results in terms of analyte sensitivity than the acetate buffer electrolyte. In both cases, well-defined signals for lead were obtained by DPSV analyses, enabling the calibration curve and figures of merit to be determined. The limits of detection (LOD) were found to be approximately 1.06 µM and 1.26 µM in H_2_SO_4_ and acetate buffer, respectively. The DPSV standard addition method was successfully applied to quantify the lead in hair dye samples, yielding values below 0.45% in Pb. All three analyzed samples were shown to comply with the limit set by the Brazilian Health Regulatory Agency, i.e., 0.6% lead in this type of product. The comparison of the electroanalytical results with those obtained by the reference method, based on the use of inductively coupled plasma optical emission spectrometry (ICP–OES), confirmed that the electroanalytical detection approach is potentially applicable as a strategy for quality control.

## 1. Introduction

Hair coloring is a fashion practice used by many people around the world. Hair salons use temporary or permanent hair dyes to give a new look to countless people; however, the process leads to the release into the sewer system of a large amount of polluted water containing residues of dyes [1]. Vegetable, synthetic and mineral hair dyes are available on the market, and dyes are distinguished by the material of manufacture [2]. Metal-based dyes are produced from metal salts, especially silver, bismuth and lead [2]. In the case of lead, the composition of hair dyes contains it in the form of lead acetate. Subsequently, by reaction with the sulfur of the keratin of the hair, a mixture of metal oxides and insoluble sulfide is formed, which is responsible for the gradual darkening of the white strands [3]. Unfortunately, lead is one of the most toxic metals [4,5] and causes a variety of environmental problems, mainly in aquatic ecosystems, for example due to bioaccumulation, oxidative stress, neurotoxicity and immune responses in fish. Due to its bio-accumulative character, once lead is absorbed, various organs of the body can be poisoned [6]. For this reason, it is necessary to monitor pollution and control exposure to lead.

Hair dyes are widely used in Brazil, and the Brazilian Health Regulatory Agency (Agência Nacional de Vigilância Sanitária, ANVISA) regulates the use of lead acetate during the production of hair dye products, limiting its concentration to a maximum of 0.6% lead [7]. In other countries, such as the United States of America, the use of lead acetate as a color additive in hair dye is currently banned [8].

There are several techniques for detecting and quantifying lead, such as inductively coupled plasma mass spectrometry (ICP–MS) [9], atomic absorption spectrometry (AAS) [10] and fluorescence [11]; unfortunately, these instrumental methods are expensive and require a whole series of operational factors aimed at optimizing each of these techniques [12]. In this scenario, an electrochemical approach would represent an ecological and economical alternative, with significant advantages, such as simplicity of operation, high sensitivity, and easy handling [9,13,14].

Electrochemical sensing has aroused great interest and is widely used for the detection of heavy metal ions and various organic compounds [15]. Different materials can be used as sensors (such as Au, Pt, Ag, Cu, Hg, and carbonaceous materials), and the selection and development of materials to modify the electrodes are important factors in improving their sensitivity and selectivity [16]. On the other hand, the choice of electrochemical methods and/or effective operating conditions for the simultaneous determination of multiple metal ions are also factors that need to be studied to improve the effectiveness of these tools [15,16,17]. For example, Zhang et al. used porous activated carbon uniformly decorated with palladium nanoparticles to modify a glassy carbon electrode (Pd@PAC/GCE) and determine traces of Cd^2+^, Pb^2+^ and Cu^2+^ ions in real samples using square-wave anodic stripping voltammetry [18], obtaining good sensitivities with respect to AAS. Differential pulse anodic stripping voltammetry and differential pulse voltammetry were used to determine Pb^2+^ in water samples (tap water, lake water and wastewater) with the application of green carbon materials, reaching limits of detection between 0.2 mg L^−1^ and 0.38 mg L^−1^, comparable or better than those obtained with instrumental methods such as ICP–MS and AAS [14,19,20].

Metal organic frameworks (MOFs) [21] and quantum dots (QDs) [22] were also investigated for the analysis of toxic metals in food and environmental applications. Radhakrishnan et al. [23] prepared a series of surface-passivated fluorescent QDs by one-pot hydrothermal methods, which were functionalized using organic probes such as L-cysteine, ethylenediamine and glycine, thereby increasing the sensitivity for Cu^2+^, Pb^2+^ and Fe^3+^ ions. Similarly, 4-(thiazolylazo) resorcinol and benzoyl isothiocyanate have shown advantages in functionalization of MOFs for lead analysis in different samples [24,25].

With this in mind, this work involved the design of an electroanalytical strategy to control the amount of lead present in progressive hair dyes using a composite cork–graphite sensor previously manufactured by our group [12]. This sensor has been shown to have a high sensitivity, a significant selectivity and a remarkable current response for the determination of different organic and inorganic substances (caffeine, paracetamol, hydroxychloroquine and lead) [12,26,27,28]. The benefits are partly due to the surface of cork, which participates as a modifier with adsorbent characteristics or by introducing further functional groups on the electrode surface, thanks to active centers such as phenolic, carboxylic, sulphonic, phosphate and amino groups [29].

As discussed in our previous reports [12,26,27,28], it is common practice to evaluate the effectiveness of an electroanalytic approach by polluting/intensifying an aqueous matrix (e.g., river water, sea water, groundwater, tap water, drinking water) with a well-known amount of a single target pollutant to understand the experimental data and translate them into real applications. However, developing a control solution to determine the amount of lead directly in real samples, without laboratory pretreatment, would be a clear benefit to our society, offering a consistent approach that is easy to handle and with low operating costs. In this context, this investigation aimed to use a composite cork–graphite sensor to directly determine and quantify lead in three commercial progressive dye samples using the DPSV standard addition method.

## 2. Materials and Methods

### 2.1. Reagents

High purity chemical reagents were used in this study. Graphite powder and Pb(NO_3_)_2_ were supplied by Vetec (Brazil), while acetate buffer and H_2_SO_4_ were sourced from Merck (Brazil). The raw cork used in the experimental studies was provided by Corticeira Amorim S.G.P.S., S.A. (Porto, Portugal). Raw cork granules were washed twice with distilled water for 2 h at 60 °C to remove impurities and other water-extractable components that could interfere with the electrochemical analysis. Before use, the raw cork was dried at 60 °C in an oven for 24 h [26]. All solutions were prepared using ultrapure water obtained from a Milli-Q (18.2 MΩ cm, 25 °C) system (Millipore).

### 2.2. Preparation of Cork-Modified Electrodes

The raw cork granules were reduced in size by means of a ball mill and sieved to obtain the finest fractions. The fraction below 150 µm (designated as raw cork powder) was selected for use in this work. The composite cork–graphite sensor (working electrode) was prepared using a mechanically homogenized mixture obtained with a borosilicate glass mortar and pestle, as reported in our previous work [12]. Raw cork powder and graphite were mixed in a proportion of 70:30% *w*/*w*; 0.3 mL of mineral oil was added to the ground powder to form a homogeneous paste, which was packed in a polypropylene nozzle (MODEL K31-200Y) used as a support. The sensor surface was finally smoothed by wiping on tissue paper. Prior to use, the sensor was electroactivated by cyclic voltammetry between −1.1 and 0.0 V (scan rate: 100 mV s^−1^) in 0.5M H_2_SO_4_.

### 2.3. Preparation of Real Samples

The hair dyes were purchased in Natal, Rio Grande do Norte, Brazil, and identified as S1, S2 and S3 solutions. For sample preparation, 1.0 g of progressive dye was weighed and diluted in 10 mL of distilled water. The mixture was homogenized, and no pretreatment was required for lead identification and quantification.

### 2.4. Electrochemical Measurements

The electrochemical analyses were performed using a three-electrode cell with an Ag/AgCl (3.0M KCl), Pt wire and a cork–graphite sensor (geometric area of about 0.45 mm^2^) as reference, auxiliary, and working electrodes, respectively, connected to an Autolab PGSTAT302N (Metrohm, Zurich, Switzerland) controlled with GPES (v. 4.0) software. To obtain the calibration curve, cyclic voltammetry (CV) and differential pulse stripping voltammetry (DPSV) measurements were performed with different concentrations of Pb(II) ions in 0.1M acetate buffer (pH 4.5) and 0.5M H_2_SO_4_ solutions.

For CV, a −0.9 V to −0.3 V potential window was cycled at 50 mV s^−1^ in the absence and presence of hair dye sample in 0.1M acetate buffer (pH 4.5) and 0.5M H_2_SO_4_ solutions. The DPSV parameters were the following: the accumulation of Pb(II) ions on the surface of the composite sensor was obtained by applying a preconcentration potential of −0.9 V (vs. Ag/AgCl) for 60 s; the last 10 s were considered as equilibration time, without stirring; modulation time: 0.04 s; interval time: 0.10 s; initial potential: −0.9 V; final potential: −0.3 V; step potential: 0.00495 V; modulation amplitude: 0.049 V; and initial stirring time: 30 s. The optimized parameters reported above were used for all measurements. All electrochemical studies were conducted at 25 ± 2 °C and without deaeration. Each measurement was performed in triplicate, and the data obtained were subjected to statistical analysis and reported as the mean ± standard deviation (SD). For the determination of Pb(II) in the different progressive hair dyes, a well-known quantity/volume of a standard solution of Pb(II) was subsequently added (standard addition method) three times, to the real sample. All of the results of the experiments were processed with GraphPad Prism v.5 and the calibrations were analyzed by ordinary linear least-square regression. The relevant results (slopes and intercepts) are reported with their confidence interval (*p* = 95%), as recommended by experts in the field [30,31,32].

### 2.5. Inductively Coupled Plasma Optical Emission Spectrometer (ICP–OES) Measurements

An ICP–OES from Thermo Fisher Scientific (Bremen, Germany, model iCAP 6300 Duo) with axial and radial view, simultaneous detector CID (Charge Injection Device) was also used to determine the concentration of the lead element in hair dye samples. Commercial argon with a purity of 99.99% (White Martins-Praxair) was used to purge the optics, for plasma generation, as well as a nebulizer and auxiliary gas. A concentric nebulizer and a cyclone-type nebulizer chamber were used in the sample introduction system. In this system, the sample is pumped into the plasma with a peristaltic pump coupled to the equipment and its flow is controlled by the software (iTeva—Thermo Scientific). The torch used was of the removable quartz type. Instrumental parameters were optimized as a function of plasma robustness for acidified aqueous solutions. The parameters were as follows: power of the frequency light source, 1350 W; nebulizer argon gas flow rate, 0.75 L min^−1^; auxiliary argon gas flow, 0.5 L min^−1^; stabilization time, 10 s.

The range of linearity was determined using a blank and 9 solutions with increasing concentrations (prepared from a 4.9 mM standard solution in 10% nitric acid, according to ISO Guide 34, AccuStandard Brand Cat. No. ICP-29N-1), which were as follows: 0.024; 0.048; 0.096; 0.19; 0.38; 0.77; 1.54; 3.09 and 6.18 µM in 10% HNO_3_. The analytical curve was prepared with high purity water (resistivity of 18.2 MΩ cm) obtained from an Elga Purelab Ultra system (Elga Labwater, High Wycombe, UK). The ICP–OES detection limit (LOD = 0.0012 mg L^−1^) was obtained from the standard deviation of 10 readings of the first point of the analytical curve, i.e., the blank, multiplied by three and divided by the slope of the curve. The limit of quantification (LOQ = 0.0036 mg L^−1^) was obtained by multiplying the LOD by three. The correlation coefficient was equal to 0.9957. The accuracy and precision were estimated from the analysis of internal (curve standards) and external (non-curve standards) solutions prepared with different concentrations and analyzed as samples, obtaining satisfactory results with errors of less than 5%, with a guarantee of more than 95% accuracy and precision.

## 3. Results and Discussion

### 3.1. Effect of the Supporting Electrolyte

**Figure 1:**
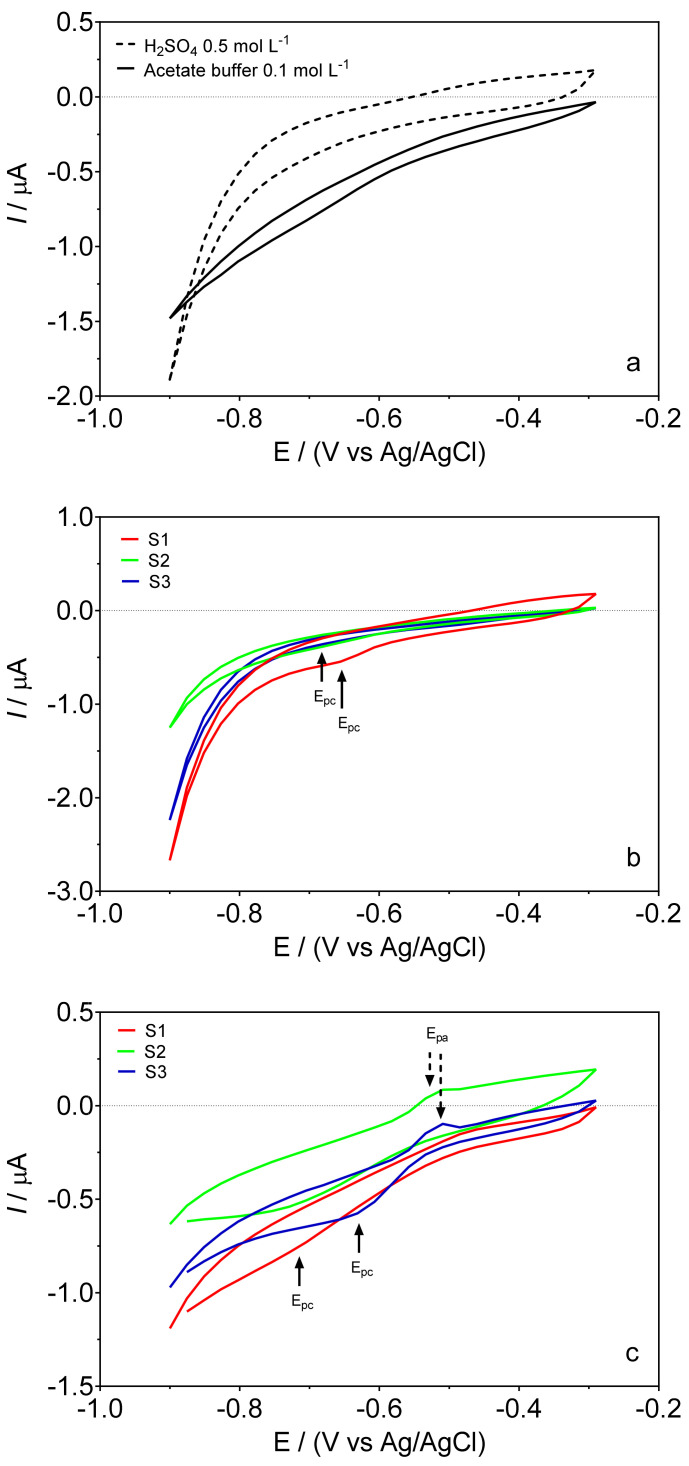
Cyclic voltammograms recorded in the absence (**a**), broken (0.5M H_2_SO_4_) and continuous (0.1M acetate buffer) (black lines) and in the presence of hair dye samples containing lead: S1 (red line), S2 (green line) and S3 (blue line) in (**b**) 0.5M H_2_SO_4_ and (**c**) 0.1M acetate buffer (pH 4.5).

**Figure 2:**
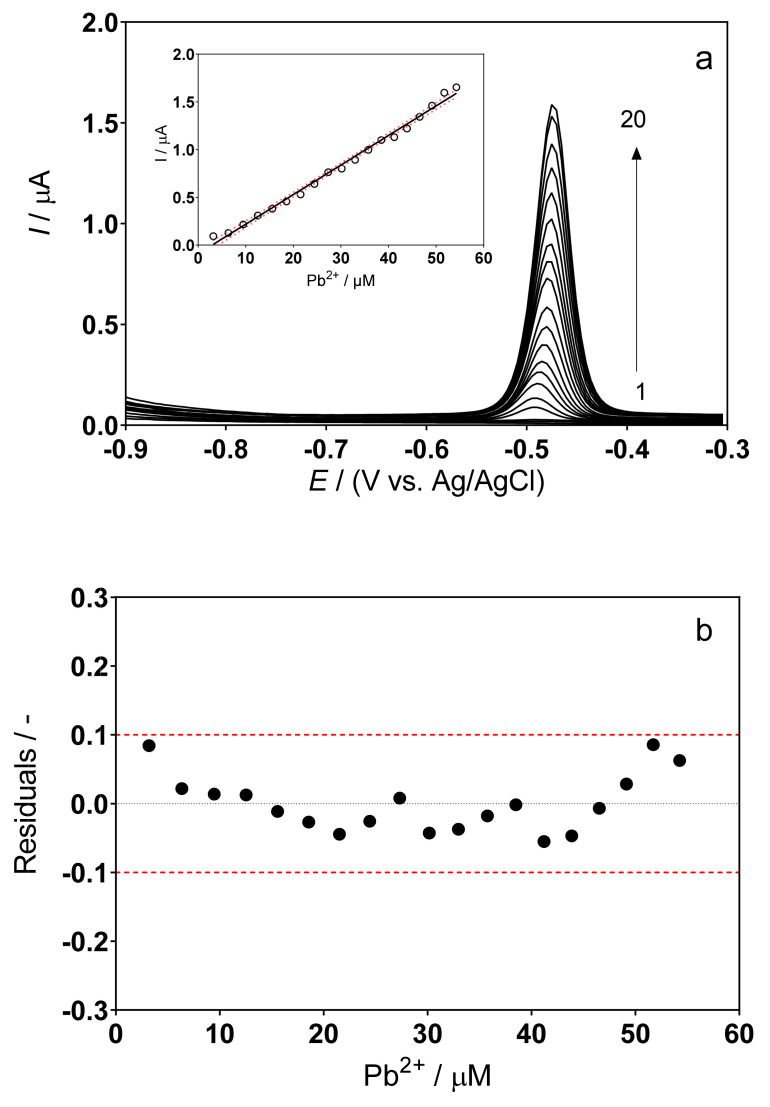
(**a**) DPSV curves recorded for different concentrations of Pb(II) in 0.5M H_2_SO_4_: (1) 0, supporting electrolyte; (2) 3.19; (3) 6.35; (4) 9.46; (5) 12.53; (6) 15.57; (7) 18.56; (8) 21.51; (9) 24.43; (10) 27.31; (11) 30.16; (12) 32.92; (13) 35.75; (14) 38.49; (15) 41.19; (16) 43.87; (17) 46.51; (18) 49.13; (19) 51.70 and (20) 54.26 µM. Inset: Plot of the electrochemical response, in terms of current, as a function of lead concentration. (**b**) Graphical representation of the residuals behavior, which confirms the linearity of the calibration curve.

Although the composite sensor has been previously evaluated [12] in terms of sensitivity, selectivity and response (using synthetic solutions) and applicability (in real samples, spiked with lead), it is known that the responses to modified electrodes strongly depend on the actual state of their electrode surface. Therefore, the voltammetric behavior of the cork–graphite composite electrode was evaluated once again in two different supporting electrolytes (0.5M H_2_SO_4_ and 0.1M acetate buffer), in the absence and presence of hair dye samples by cyclic voltammetry (Figure 1) and differential pulse voltammetry (Figure 2). Figure 1a shows that the behavior of the composite sensor in the chosen supporting electrolytes, in the absence of hair dye in solution, was dependent on the pH conditions and changes in permeability of the electrode surface. Overall, the voltammograms of the cork–graphite sensor showed a low current background in both electrolytes, showing no significant current signals related to anodic and cathodic peaks. However, the behavior of each hair dye sample (S1, S2 and S3) was also studied by CV to understand the cork–graphite surface response by adding a known volume of S1, S2 and S3 to the supporting electrolyte (Figure 1b,c). The cyclic voltammograms obtained at the lower concentrations of S1, S2 and S3 showed no significant changes in the profile (data not shown). However, when the volume of S1, S2 and S3 additions increased, the composite sensor exhibited notable peak responses in the potential range investigated. As can be observed in Figure 1b, the voltammograms did not show any anodic peak, while a single cathodic peak was observed at ca. −0.65 V (so-called E_pc_) for S1 and at ca. −0.68 V for S2 and S3 in 0.5M H_2_SO_4_. Conversely, anodic peak (so-called E_pa_) signals were registered for S2 and S3 at approximately −0.51 V in the case of measurements performed in 0.1M acetate buffer (Figure 1c), while the cathodic peak signal was observed at ca. −0.69 V for S1 and S2 samples and at ca. −0.63 V for S3. In both supporting electrolytes containing hair dye samples, the cathodic peak can be confidently assigned to lead detection, also in agreement with what other authors have seen, who have assigned the ion current signal between −0.56 and −0.68 V in the cathodic scan [14,33,34] to the reduction of lead ions (Equation (1)).

Pb^2+^ + 2e^−^ → Pb_(s)_ (1)

Regarding the anodic peak recorded in the CV profiles of the S2 and S3 samples in acetate buffer (Figure 1c), it could be associated with the interference of compounds present in the hair dye samples and reported by the manufacturer, such as ammonium, propylene glycol, dyes and other salts. The oxidation of these compounds on the surface of the cork–graphite sensor is related to the change in pH conditions, which affects the chemical structure of organic substances and salts in solution, as well as the active sites of cork, which are not observed at acidic conditions [14,26]. However, the oxidation peaks did not interfere in the reduction of lead ions. In fact, the experimental observations show that during the cathodic scan, a strong surface interaction is obtained between the lead ions present in the solution and the cork–graphite surface, mainly due to the active centers made available by the cork [12,14,28,35,36]. Hence, this cathodic voltammetric signal can be used for the quantification of lead in the samples.

Based on the recommendations of the experts [37,38,39] and the previous results reported in [12], the DPSV determination of traces of lead should be investigated once again, evaluating the actual state of the composite sensor with respect to the affinity to lead and obtaining analytical curves in both supporting electrolytes, in the absence and presence of the hair dye in solution.

### 3.2. DPSV Determinations: Effect of the Supporting Electrolyte

**Figure 3:**
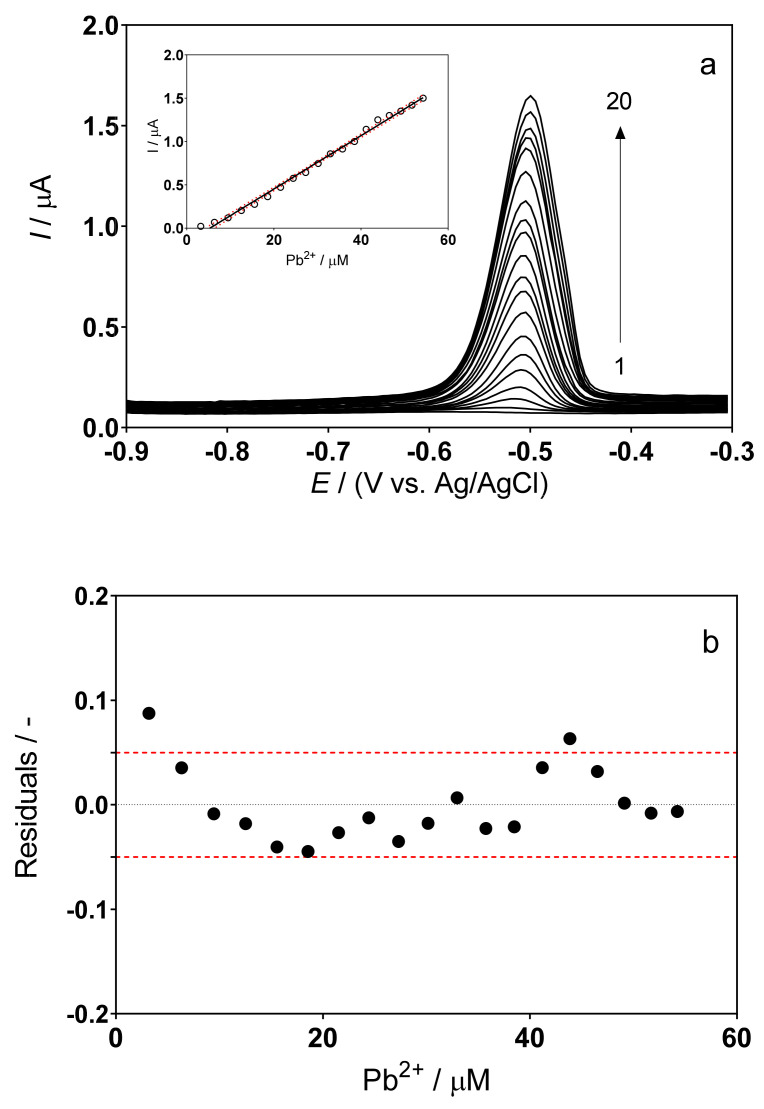
(**a**) DPSV curves recorded for different concentrations of Pb(II) in 0.1M acetate buffer (pH 4.5): (1) 0, supporting electrolyte; (2) 3.19; (3) 6.35; (4) 9.46; (5) 12.53; (6) 15.57; (7) 18.56; (8) 21.51; (9) 24.43; (10) 27.31; (11) 30.16; (12) 32.92; (13) 35.75; (14) 38.49; (15) 41.19; (16) 43.87; (17) 46.51; (18) 49.13; (19) 51.70 and (20) 54.26 µM. Inset: Plot of the electrochemical response, in terms of current, as a function of lead concentration. (**b**) Graphical representation of the residuals behavior, which confirms the linearity of the calibration curve.

The DPSV responses for the determination of Pb(II) using the cork–graphite sensor in 0.5M H_2_SO_4_ and 0.1M acetate buffer (pH 4.5) solutions, respectively, in the absence of the hair dye in solution are shown in Figure 2 and Figure 3.

In both electrolytes, the composite cork–graphite sensor provided good current responses in the presence of lead ions. However, the H_2_SO_4_ solution proved more suitable, as it provided a well-defined voltammetric signal and the response increased linearly with no significant deviations (Figure 2a). In the case of the acetate buffer, a slight increase in peak width was observed at higher lead concentrations. Although this effect did not significantly affect the response to lead concentration, it must nevertheless be associated with interactions of the cork–graphite surface with the lead species in solution.

As previously reported [14,26,28,40], the voltammetric current signals, in terms of length, width and height, can be enhanced when cork is used as a modifier. This is due to a bio-absorption mechanism in which the initial physical adsorption (rapid metal entrapment) is followed by slow chemisorption. Additionally, this mechanism can be enhanced depending on the pH conditions and the type of cork used [14] because specific active sites (phenolic, carboxylic, sulphonic, phosphate and amino groups, as well as coordination sites) may predominate in the composition of the surface [41]. On the other hand, the use of graphite contributes to the morphology of the surface by positively influencing the current response, as evidenced through scanning electron microscopy images [26,35].

As can be seen in the insets of Figure 2a and Figure 3a, under the experimental conditions described above, a linear correlation interval was obtained between the peak current and the concentration of metal ions. The data obtained from the analyses of standard solutions in the chosen medium allowed the estimation of the functional relationship (peak current vs. concentration), which is linear in the range from 3.19 to 54.26 µM. It is important to note that, at lower concentration values, the reproducibility of the acetate buffer response was poorer.

The calibration curves, shown in the insets of Figure 2a and Figure 3a, made it possible to derive the following equations:

H_2_SO_4_: → I/µA = (0.0311 ± 0.03) × [Pb^2+^] − (0.091 ± 0.76) × 10^−6^ → (α = 0.05, *n* = 20, r^2^ = 0.99)

Acetate buffer: → I/µA = (0.0308 ± 0.05) × [Pb^2+^] − (0.163 ± 0.54) × 10^−6^ → (α = 0.05, *n* = 20, r^2^ = 0.99)

Since all analyses were performed in triplicate, it was possible to obtain confidence intervals and standard deviations within 95% (red dotted lines in the analytical curves, insets in Figure 2a and Figure 3a). This information was used to identify false positives and false negatives (α = β = 0.05), as already reported by experts in the field [30]. Figure 2b and Figure 3b show that the residuals of the regression are randomly distributed around zero, allowing a visual verification of the absence of significant non-linearities [30,31].

The limit of detection (LOD) and limit of quantification (LOQ) were found to be 1.06 µM and 4.32 µM for the H_2_SO_4_ solution and 1.26 µM and 3.29 µM for the acetate buffer, according to Equations (2) and (3), where, according to IUPAC, S_y/x_ is the residual standard deviation and *b* is the slope of the calibration plot [30,32,42,43]:

LOD = 3.3 × S_y/x_/*b* (2)

LOQ = 10 × S_y/x_/*b* (3)

For both electrolytic media, the estimated LOD values are similar, indicating that both electroanalytical strategies could be implemented in real matrices. However, as previously indicated, the effectiveness of a given technology is usually evaluated using ultrapure/demineralized water with a single target pollutant and/or at a concentration several orders of magnitude higher than the real environment or real samples. Therefore, it is recommended to supplement the work with experimental data obtained using real matrices and suitable contaminant concentrations (in line with the expected concentrations) when these ‘real’ samples are created in the laboratory. The standard addition method is useful for avoiding matrix effects and has been used to electrochemically quantify lead in real hair dye samples.

Table 1 collects the results available in the literature and related to the electrochemical analysis of Pb(II) with different sensors, including the present work and LOD values obtained for both supporting electrolytes. As can be seen, the LOD values achieved in the present work are higher than most of those obtained with other electrodes; however, the applicability conditions of the sensor should also be considered (e.g., lead concentration to be detected and matrices to be analyzed) and the need for modification approaches to improve the sensor response.

The sensor proposed here is a quality control tool suitable for validating the quantity of lead present in hair dye products, although it could also be used as an environmental sensor to control the amount of lead released into the environment when hair coloring effluents are produced and discharged. Since the proposed sensor preparation does not involve toxic or expensive materials such as nanotubes, nanoparticles, polymer films or mercury polarography (see Table 1), this sensor represents a more cost-effective strategy than other sensors and other instrumental analyses. Therefore, the LOD values obtained are suitable for the proposed application, and the next step is to test the reliability of the proposed sensor to detect lead in real samples.

### 3.3. Determination of Lead Concentration in Hair Dye Samples

**Figure 4:**
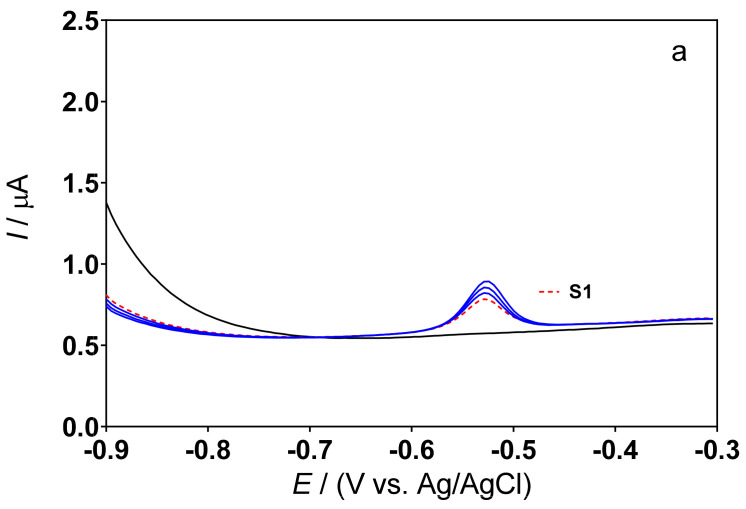

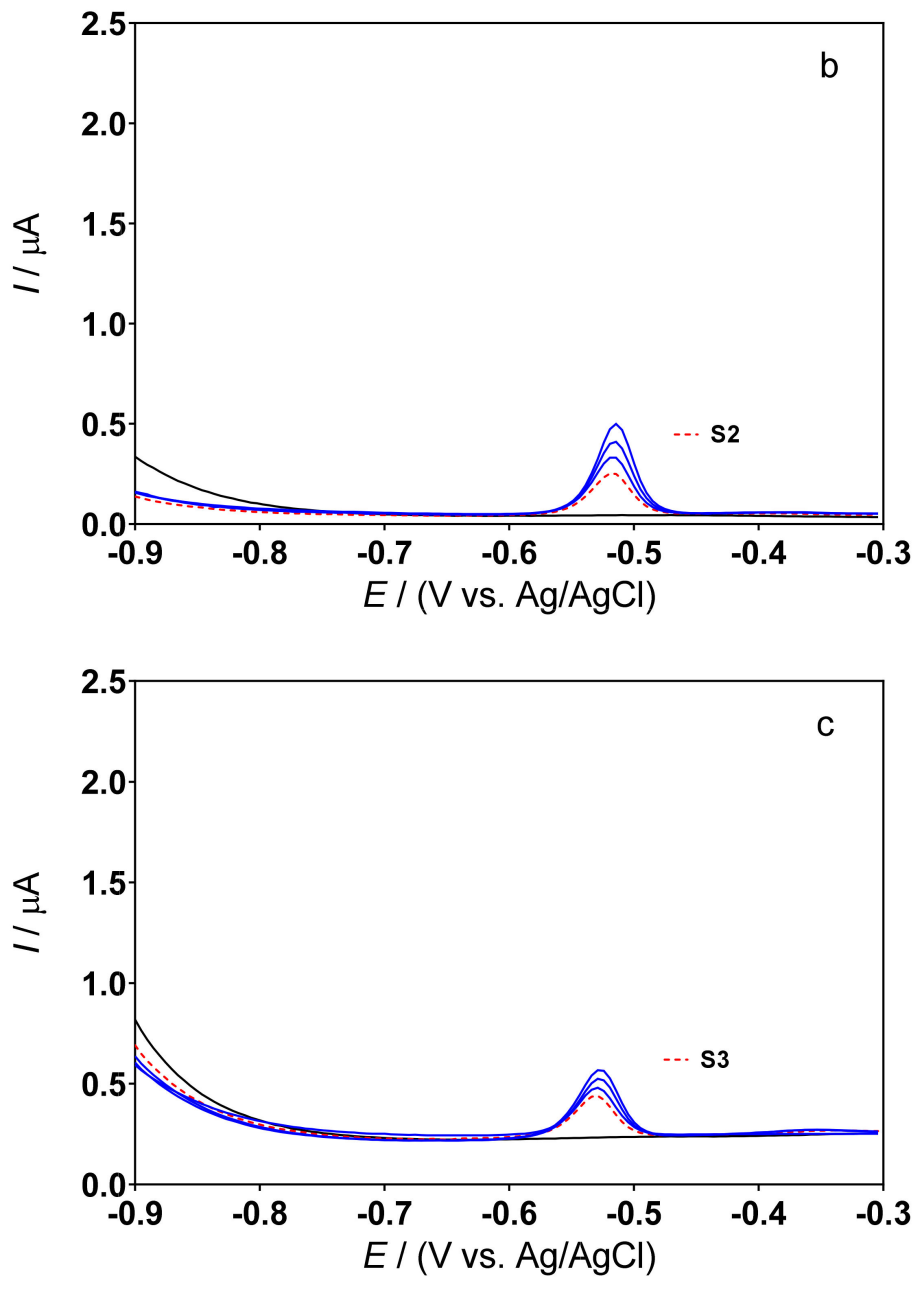
Results of the determination of Pb(II) in different samples (**a**: S1; **b**: S2, and **c**: S3) by means of the standard addition method. DPSV profiles obtained in the supporting electrolyte (black line) and in the hair dye sample (dashed red line) without any addition of Pb, as well as profiles obtained after the additions of 100, 200 and 300 µL of a standard solution of 100 mg L^−1^ Pb(II) in 0.5M H_2_SO_4_ (blue lines).

**Figure 5:**
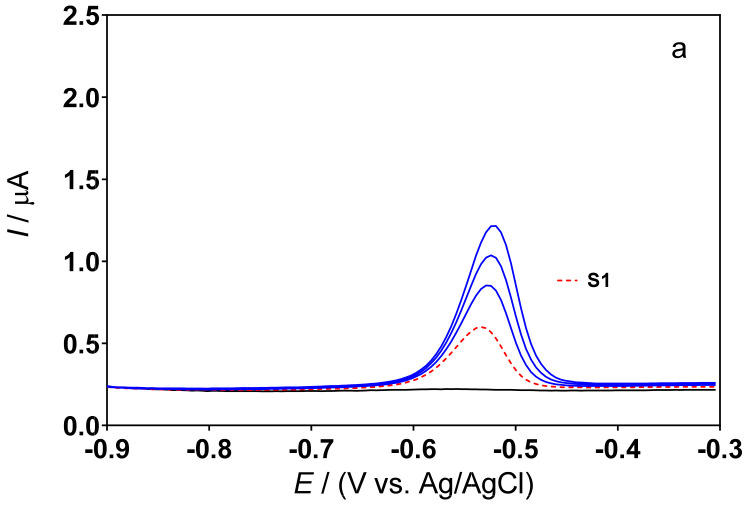

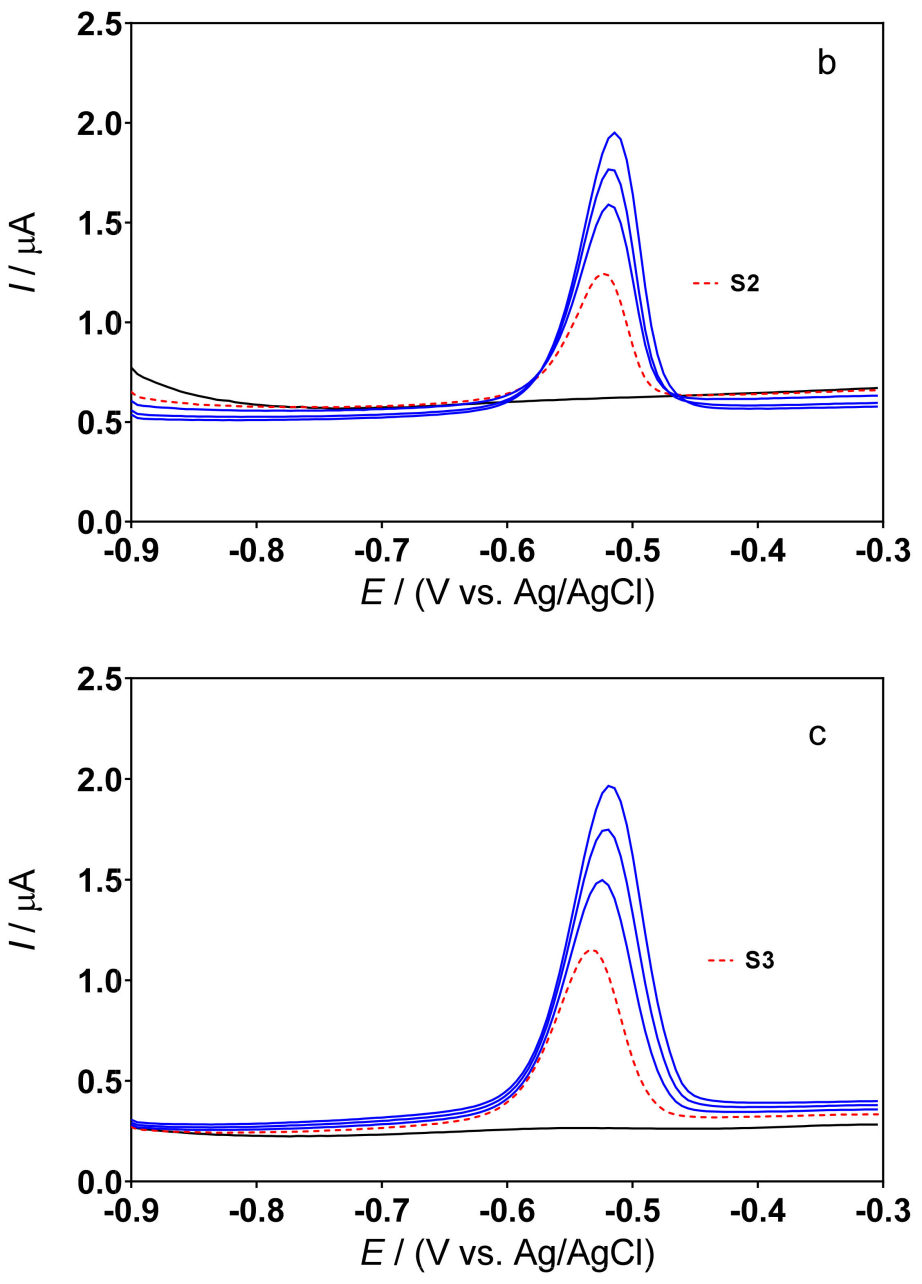
Results of the determination of Pb(II) in different samples (**a**: S1; **b**: S2, and **c**: S3) by means of the standard addition method. DPSV profiles obtained in the supporting electrolyte (black line) and in the hair dye sample (dashed red line) without any addition of Pb, as well as profiles obtained after the additions of 100, 200 and 300 µL of a standard solution of 100 mg L^−1^ Pb(II) in 0.1M acetate buffer (blue lines).

To investigate the suitability of the approach for environmental Pb limit controls, the new low-cost and green sensor was finally used to measure Pb(II) concentrations in three progressive hair dye samples (S1, S2 and S3). Known amounts of Pb(II) were added (standard additions of 100, 200 and 300 µL of a solution containing 100 mg L^−1^ of Pb(II) in 0.5M H_2_SO_4_ or 0.1M acetate buffer (pH 4.5)) to all samples (S1, S2 and S3). As can be seen in Figure 4 and Figure 5, no current signal was obtained, in all cases, when the supporting electrolytes were analyzed in the absence of a hair dye sample (solid black line (—). Instead, a well-defined peak was recorded around −0.5 V when the hair dye sample was added to the supporting electrolyte (dashed red line (**---**)).

By comparing the data obtained with the analytical curves of Figure 2a and Figure 3a, the signals recorded by analyzing samples S1, S2 and S3 appear to correspond to Pb(II), and indicate its presence in each of the real samples examined electroanalytically. Furthermore, the presence of lead was confirmed by the intensification of the peak following the addition of different volumes of standard solution (Figure 4 and Figure 5). It is interesting to note that no interferences appeared due to the presence of other substances in the samples, as also commented on previously in the case of the investigation of contaminated synthetic samples [12]. In all cases, the peak current increases linearly with each intensifying addition, and the intensity of the peak responses, in terms of current, changes depending on the electrolyte used (0.5M H_2_SO_4_, Figure 4, or 0.1M acetate buffer, Figure 5). However, more defined and intense peaks were recorded in the acetate buffer than those obtained in the sulfuric acid. Based on the data obtained, the composite cork–graphite sensor can be effectively used to determine the Pb(II) concentration in hair dye samples using the DPSV standard addition method. Additionally, sensor stability was assessed by DPSV as well as interday and intraday repeatability, obtaining reliable results.

Table 2 reports the Pb(II) concentration determined in all samples using the proposed sensor as well as the instrumental reference method: the Pb(II) values obtained with both techniques, ICP–OES and DPSV, do not appear too dissimilar. ICP–OES is a reference technique for the detection of heavy metals; however, it has a significantly higher operating cost than the proposed electroanalytical approach. The mean results were obtained by recording three measurements with acceptable standard deviations and confidence intervals relating to a probability of 95%. This approach allows the verification of both false positives and false negatives (α = β = 0.05), as recommended by the IUPAC [42,51].

As regulated by ANVISA, a maximum limit of 0.6% of Pb in cosmetic compounds is allowed; therefore, all samples appear in compliance with the Brazilian legislation. Based on the data obtained (Table 2), the determination of Pb(II) in commercial hair dye samples using a composite sensor allows excellent performance in terms of reliability, sensitivity and quantification compared to the reference method, ICP–OES, which confirms that the electroanalytical approach developed is potentially applicable as a quality control strategy.

## 4. Conclusions

The DPSV approach using cork–graphite-based sensors offers a quick, reliable, cost-effective and simple way to determine Pb(II) in hair dye samples. The composite sensor has sufficient sensitivity and reproducibility, and the low LOD allows for the minimization of matrix effects in dilute solutions using the DPSV standard addition method. The proposed approach is precise, with an LOD of 1.06 µM in 0.5M H_2_SO_4_ and of 1.26 µM in 0.1M acetate buffer. The electroanalytical strategy is reproducible and less expensive, both in terms of time and materials, than other analytical methods.

## Figures and Tables

**Table 1:** Comparison of the analytical parameters of the sensors reported in the literature for the determination of Pb(II).

Electrodes	Samples	Method	Electrolyte	Linear Range (µM)	Limit of Detection (LOD, µM)	Ref.
^1^ CPME-ACfB300	Hair dyes and gunshot residues	^2^ DPAdSV	0.1M acetate buffer (pH = 7.0)	0.13–2.44	0.0045	[44]
^3^ CB-CP	Hair dyes	^8^ DPV	0.2M phosphate buffer (pH = 5.0)	0.048–0.48	–	[4]
Disposable plastic electrode	Cosmetic samples	DPSV	0.8M HNO_3_—0.1M KCl	0.24–0.72	0.096	[45]
^4^ GCE	Hair dyes	^9^ ASV	0.1M NaCl	7.0–17.5	0.021	[46]
^5^ AgNF-Pb@GCE	Cosmetic samples	^10^ SWV	Acetate buffer (pH 4.5)	0.4–3.3	0.035	[47]
^6^ AgSPE	Cosmetic hair dye	SWV	0.05M Na_2_SO_4_	0.06–0.79	0.031	[48]
^7^ CW-ISE	Cosmetic samples	Potentio-metric method	Acetate buffer (pH 5.0)	10–100	8.0	[49]
Graphite/epoxy composite electrodes	Hair cosmetic	^11^ SWASV	0.1M acetate buffer (pH 6.0)	0.2–1.7	0.07	[50]
Cork-graphite	Hair dyes	DPSV	0.5M H_2_SO_4_	3.19–54.26	1.06	This work
Cork-graphite	Hair dyes	DPSV	0.1M acetate buffer (pH 4.5)	3.19–54.26	1.26	This work

^1^ Carbon paste electrode containing chemically treated biochar; ^2^ differential pulse adsorptive anodic stripping voltammetry; ^3^ carbon black-modified carbon paste sensor; ^4^ gold nanoparticle (AuNP)/hexaammineruthenium(III) ([Ru(NH_3_)_6_]^3+^)/Nafion^®^ modified glassy carbon electrodes; ^5^ silver nanoflower modified glassy carbon electrode; ^6^ screen-printed silver electrodes; ^7^ coated wire ion selective electrode; ^8^ differential pulse voltammetry; ^9^ anodic stripping voltammetry; ^10^ square wave voltammetry; ^11^ square wave anodic stripping voltammetry.

**Table 2:** Pb(II) concentration in hair dye samples determined by DPSV and ICP–OES. % values in the samples and % difference between the two control determinations.

Sample	Method	[Pb^2+^]/mg L^−1^	Pb Content (%)	Difference (%) ***
S1	ICP–OES	4.14 ± 1.02	0.25	
	DPSV *	4.00 ± 1.65	0.20	3.4
	DPSV **	3.89 ± 1.90	0.18	6.0
S2	ICP–OES	2.42 ± 0.58	0.30	
	DPSV *	2.33 ± 0.23	0.26	3.7
	DPSV **	2.47 ± 0.41	0.32	2.1
S3	ICP–OES	3.50 ± 0.39	0.43	
	DPSV *	2.85 ± 0.25	0.34	18.5
	DPSV **	2.65 ± 0.48	0.30	24.3

* Standard addition method using the DPSV approach with cork–graphite sensor in 0.5 M H_2_SO_4_; ** Standard addition method using the DPSV approach with cork–graphite sensor in 0.1M acetate buffer; *** |% Difference| = 100 × (DPSV − (ICP–OES))/(ICP–OES).
